# A strategy for the treatment of gastrointestinal cancer: Targeting tumor senescent cells

**DOI:** 10.3389/fmolb.2023.1139840

**Published:** 2023-03-06

**Authors:** Yujing Liu, Qiang Zhang, Wenjing Ni, Guang Ji, Hanchen Xu

**Affiliations:** ^1^ Institute of Digestive Diseases, Longhua Hospital, Shanghai University of Traditional Chinese Medicine, Shanghai, China; ^2^ Department of Digestive Endoscopy, Jiangsu Province Hospital of Traditional Chinese Medicine, Affiliated Hospital of Nanjing University of Chinese Medicine, Nanjing, Jiangsu, China; ^3^ Shanghai Frontiers Science Center of Disease and Syndrome Biology of Inflammatory Cancer Transformation, Shanghai, China

**Keywords:** gastrointestinal (GI) cancer, cell senescence, cancer therapy, age-related secretory phenotype (SASP), aging markers

## Abstract

Gastrointestinal (GI) cancer includes a variety of cancers with high incidence that seriously threaten the lives of people worldwide. Although treatment strategies continue to improve, patient benefits are still very limited, and the ongoing search for new treatment strategies remains a priority. Cell senescence is closely related to the occurrence and development of tumors. For GI cancer, cell senescence may not only promote cancer but also bring new opportunities for treatment. Combined with relevant studies, we review the dual role of cell senescence in GI cancer, including the mechanism of inducing cell senescence, biomarkers of senescent cells, and potential of targeted senescence therapy for GI cancer.

## 1 Introduction

Cell senescence is permanent cell cycle arrest associated with a variety of secretory phenotypes. Induction of cell senescence is an important mechanism of tumor inhibition by chemoradiotherapy and targeted therapy (Gomes et al., 2020). However, as a self-protective mechanism of cells, senescence has both advantages and disadvantages in tumor therapy (Song et al., 2022). GI cancer mainly includes a variety of malignant tumors, which are difficult to treat in the clinic. A new approach to treat GI cancer is the use of the tumor suppressive effect of cellular senescence, avoiding the tumor promoting effect, improving the sensitivity of treatment, and preventing tumor recurrence and metastasis. The purpose of this paper is to review the “double-edged sword” effect of cell senescence in the treatment of GI cancer and explain the specific role of cell senescence in the process of GI cancer, and the potential mechanism of targeting senescent cells in the treatment of GI cancer are also discussed.

## 2 Major changes after cell senescence

**FIGURE 1:**
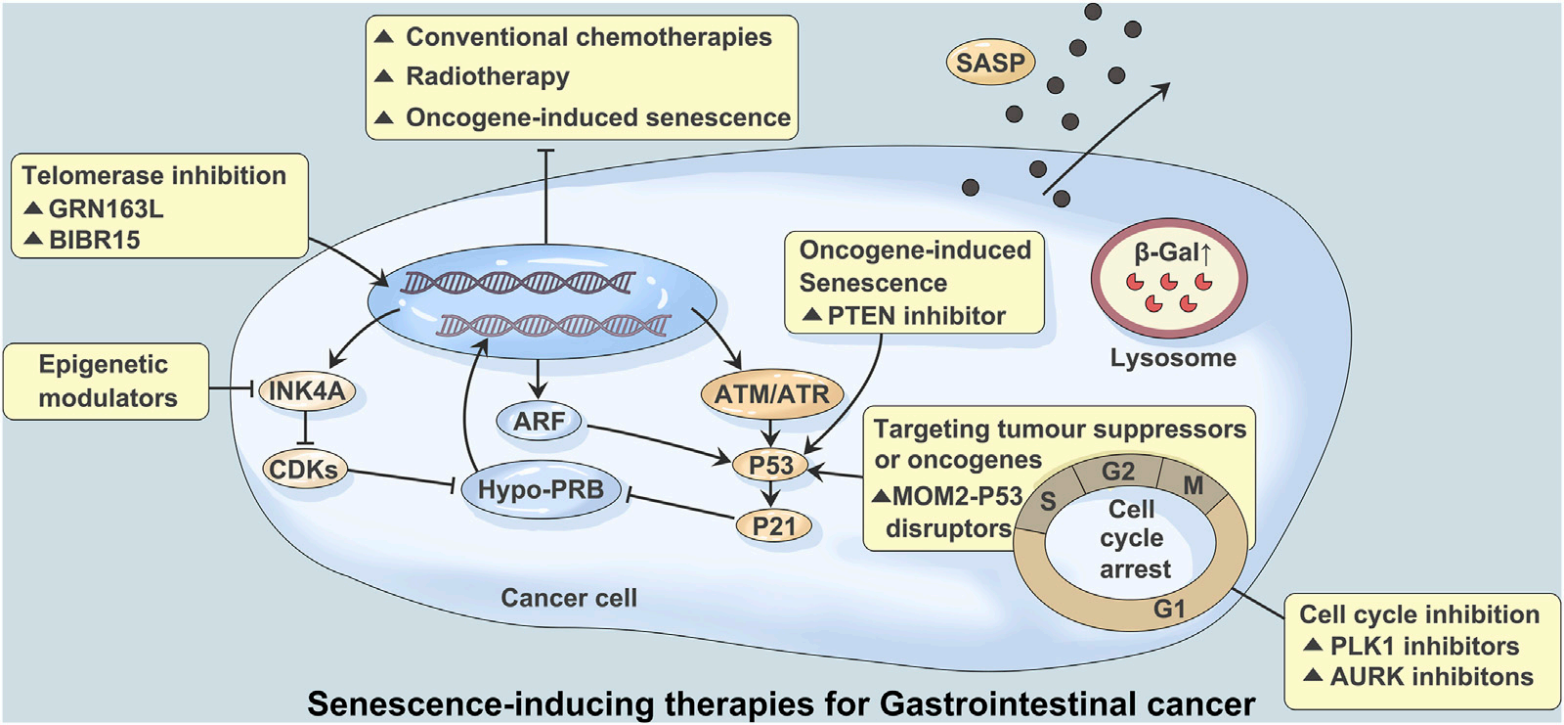
Senescence-inducing therapies for Gastrointestinal cancer. Studies have shown that the mechanisms regulating tumors and aging mainly involve mitochondrial replacement, genome editing, telomere maintenance and immune editing. Persistent DNA damage caused by over-activation of cancer-promoting signals, conventional chemoracial therapy, and treatment with telomerase inhibitors (GRN163L/BIBR15) often leads to induction of aging. A large number of DNA damage responses trigger ATM or ATR signaling and lead to activation of p53 and p21. Aging mediated by p53-p21 signaling can also be triggered by PTEN inhibitors. Inhibition of mediated aging by activation of p53-mediated and cyclin dependent kinases (CDK) *via* transcripts encoding ARF and INK4A. CDK inhibition mediated aging can be achieved through CDK4 and CDK6 inhibitors. Aurora kinase (AURK) and PLK1 inhibitors block the G2/M process of the cell cycle, which can also induce aging. At the same time, stable cycle arrest, *β*-Galactosidase (*β*-Gal) activation, and age-related secretory phenotype (SASP) production were observed.

Aging is a process of gradual loss of physiological integrity, resulting in functional impairment and gradual death (Hill et al., 2020). Aging is characterized by genomic instability, telomere shortening, epigenetic changes, loss of protein homeostasis, sensitivity to dystrophy, mitochondrial dysfunction, cellular senescence, stem cell failure, and altered intracellular signaling (Calcinotto et al., 2019) (Figure 1). Recent studies have found that cellular senescence, one of the characteristics of aging, as a response to endogenous and exogenous stress, is not only closely related to age-associated diseases and tissue ageing, but also plays an important role in tissue repair and tumorigenesis (Shmulevich and Krizhanovsky, 2021). Therefore, identifying key features of cell senescence and targeting tumor cell senescence is a promising emerging research field in tumor therapy (Wang et al., 2022).

Unlike dormant cells, senescent cells have some common aging phenotypes and characteristics in addition to growth stagnation. These features include flat, enlarged morphology, endoplasmic reticulum stress, increased mitochondrial number, and anti-apoptosis, among others (Calcinotto et al., 2019). Although senescent cells are in growth arrest, they still have relatively active metabolic activity, synthesizing many proteins, such as interleukin, growth factors, chemokines, and matrix metalloproteinases, and affecting the tissue microenvironment in the form of paracrine signaling. This signature feature is known as the age-related secretion phenotype (Gomes et al., 2020). SASP has important biological functions in wound healing and tissue microenvironment remodeling and in promoting tumor proliferation and metastasis (Birch and Gil, 2020). Thus, induced cellular senescence may represent an effective cancer treatment (Blasiak, 2020). Another characteristic of cellular senescence is upregulation of some lysosome proteins and an increase in lysosome content, among which beta-galactosidase related to lysosome aging is widely used as the most common marker of aging in the detection of cellular senescence (Levine and Kroemer, 2019).

## 3 Cellular senescence: A “double-edged sword” in cancer therapy

Cellular senescence may have different intervention effects on cancer through cellular autonomous and cellular involuntary mechanisms (Saleh et al., 2018). Cellular autonomic mechanism refers to the overactivation of intracellular oncogenes and the inactivation of tumor suppressor genes or various SASP factors produced by senescent cells through autocrine action on tumor cells, thereby influencing cell senescence (Di Mitri and Alimonti, 2016). Cellular involuntary mechanisms refer to the way in which senescent cells regulate other cellular components around them through paracrine signaling (Herranz and Gil, 2018; Saleh et al., 2018). Therefore, we reviewed the “double-edged sword” effect of cell senescence on tumor therapy from the aspects of autonomous and involuntary mechanisms of tumor cells.

### 3.1 Mechanisms and effects of cell autonomy

#### 3.1.1 Antitumor effects caused by cell-autonomous mechanisms

SASP, an important characteristic of senescent cells, can affect the effectiveness of cancer treatment (Rao and Jackson, 2016; Narita and Narita, 2017). SASP not only antagonizes tumor cells through autocrine effect, but also acts on tumor microenvironment (TME) through paracrine effect, causing different biological effects (Collado, 2010; Bian et al., 2012; Zhou et al., 2018). Recent studies have shown that Sesn3 plays a tumor suppressor role by modulating signal transduction and transcriptional activator 3 (STAT3) signaling pathways (Liu et al., 2019). The STAT3 pathway is closely associated with HCC progression, and phosphorylation at Tyr705 of this pathway leads to significant enrichment of the MMP-9 gene promoter (Jia et al., 2017). In addition, riboophorin II (RPN2) induces the expression of MMP-9 through the STAT3 and NF-kB pathways, thereby promoting hepatocellular carcinoma metastasis (Bi and Jiang, 2018; Huang et al., 2019). Studies have shown that heterochromatin protein family 1 (HP1), such as heterochromatin protein 3 (CBX3), blocks cell cycles and promotes the occurrence, development, invasion and metastasis of hepatocellular carcinoma by regulating downstream genes such as p21, cyclin dependent kinase 6 (CDK6) and CD44 (Wang et al., 2021). In addition, activation of the insulin-like growth factor (IGF) signaling pathway leads to tumorigenesis in a variety of cancers, including liver cancer, and insulin-like growth factor binding protein 7 (IGFBP7) induces cancer-specific cellular senescence by inhibiting this signaling pathway (Akiel et al., 2017; Karagiannis et al., 2019; Kotsantis et al., 2019).

#### 3.1.2 Tumor-promoting effects due to cell-autonomous mechanisms

Studies have shown that senescence induced by chemotherapy and radiotherapy enables cancer cells to acquire stem cell characteristics. This process is closely related to the Wnt signaling pathway, p53, p21, etc. (Butera et al., 2018; Zhong et al., 2019) (Figure 1). Moreover, long-term culture of senescent cells is a potential risk factor for tumor recurrence and metastasis due to the high complexity of the surrounding microenvironment (Wang et al., 2021). Indeed, studies have found that a small number of senescent cells may recover their proliferative characteristics during long-term culture, thereby promoting tumor progression (Marongiu et al., 2014; Marongiu et al., 2016; Zhou et al., 2017). The complexity of SASP factors is an important reason for the “double-edged sword” effect of SASP. How to make use of the antitumor effect of SASP and avoid its possible protumor effect are an important problem that have not been solved in this field.

### 3.2 Cellular non-autonomous mechanisms and effects

#### 3.2.1 Antitumor effects caused by non-cell-autonomous mechanisms

Studies have shown that SASP can induce the senescence of adjacent tumor cells through paracrine, maintain the senescence state of cells, and exert tumor inhibition effect (Mossanen et al., 2019). Observed the expression of NRAS proto-carcinogen in mouse hepatocytes and found that senescence hepatocytes directly secrete some cytokines, including interleukin (IL)-1α, monocyte chemotactic protein-1 (MCP1) and skin T cell attraction chemokine (CTACK) (Loo et al., 2017). Inflammatory cells such as CD4 + T cells, monocytes and macrophages (Kupffer cells) were recruited to eliminate precancerous liver cells and inhibit the occurrence of liver cancer. In conclusion, SASP factors can interact with immune cells in the tumor microenvironment and become targeted drugs for specific anti-tumor therapy (Kang et al., 2011; Lee et al., 2017; Mossanen et al., 2019; Prasanna et al., 2021).

#### 3.2.2 Tumor-promoting effects caused by cell-nonautonomous mechanisms

SASP plays a very complex role in the process of tumorigenesis. In addition to inhibiting cancer, SASP can also partly promote cancer. This is closely related to the secretion and production of cytokines and chemokines that alter the tumor microenvironment (Zhou et al., 2017). Studies have shown that senescence tumor-associated fibroblasts (CAFs) can synthesize and secrete large amounts of IL-6 and other SASP factors, thus promoting the invasion and metastasis of pancreatic cancer (Yamao et al., 2019). On the other hand, epithelial-mesenchymal transformation (EMT) is involved in tissue remodeling, trauma recovery, and the regulation of embryonic development *in vivo*. SASP factor can promote tumor migration and metastasis by promoting EMT (Ortiz-Montero et al., 2017). Studies have shown that senescent cells release SASP factors such as IL-6 and IL-8 into the tumor microenvironment, which can induce epithelial-mesenchymal transformation and thus play a role in promoting metastasis and invasion (Coppe et al., 2008). Matrix metalloproteinases (MMP) are components responsible for the breakdown and reconstruction of extracellular matrix and basement membrane that promote tumor metastasis (Falk et al., 2018). It was found that senescence hepatocytes regulate and enhance the expression of matrix metalloproteinases through nuclear factor (NF)-κB signaling pathway. This suggests that cell senescence may be involved in the invasion and metastasis of liver cancer by up-regulating the expression of MMP.

## 4 Mechanism of inducing senescence of tumor cells

Senescence and tumorigenesis have similar biological bases; that is, the abnormal mitochondrial function of tumor cells is consistent with the decline in mitochondrial function during senescence. Since tumors and senescence have a common mechanism, regulation of tumor cells and senescence can treat tumors and achieve antisenescence. Recent studies have shown that the mechanisms regulating tumors and senescence mainly involve mitochondrial replacement, genome editing, telomere maintenance and immune editing (Yu et al., 2020) (Figure 1).

### 4.1 Mitochondrial replacement

Mitochondria play an important role in the process of tumorigenesis and senescence. Replacement of defective or dysfunctional mitochondria undoubtedly resists aging of the body. The reconstruction of fat1 function by gene editing may also regulate mitochondria and indirectly control tumor and senescence (Song et al., 2022).

### 4.2 Genome editing

CRISPR‒Cas9 RNA-mediated DNA endonuclease has led to new breakthroughs in the life sciences, enabling gene editing in living cells based on cluster-separated short palindromic repeats. It is conceivable that genes regulating the growth of tumor cells can be integrated into senescent cells by genome editing technology to resist senescence. On the other hand, incorporating genes that control senescence into tumor cells can cause them to age. In addition, cellular or gene therapies, including bone marrow suppression and gene recombination, can inhibit immune senescence (Faget et al., 2019).

### 4.3 Telomere maintenance

Telomeres maintain the stability of normal cell genomes, but the gradual shortening of telomeres during cell division induces chromosome instability. Gene mutation may activate telomerase, reconstructed telomerase promotes tumor occurrence and development, and telomerase inhibitors have become the target of precision tumor therapy. Approximately 10% to 15% of human cancers maintain telomere length through homologous DNA repair *via* telomere-lengthening replacement mechanisms. Tumor cells use a special replication for DNA breaking-induced telomere synthesis (Prasanna et al., 2021) (Figure 1).

### 4.4 Immunoediting

Immunosenescence is characterized by cell-mediated immune decline, age-related humoral decline, and age-dependent T cell and B cell dysfunction. Natural killer (NK) cells are congenital cytotoxic immune cells that specifically kill tumor cells and virus-infected cells. Immunosenescence of NK cells is manifested by low expression of activated receptors in subsets of NK cells, leading to reduced cytotoxicity, and NK cells with low expression of activated receptors are common in tumor patients. Re-editing the immune system to reverse the body’s immune senescence can not only resist senescence but also be an effective way to treat tumors (Prieto and Baker, 2019).

## 5 The role of senescence markers in GI cancer

### 5.1 p53 and phosphorylated p53

Colorectal cancer is the cancer entity with the highest prevalence of p53 mutations, with 43% of CRCs carrying p53 mutations. As a major player in the DNA damage response (DDR) pathway, p53 is also a key regulator of the cell cycle, whereby increased phosphorylation of p53 activates cyclin-dependent kinase inhibitors (CDKIs) and ultimately leads to cell cycle arrest. In a mouse tumor model, deletion of the p53 mutant gene slows cancer growth and prolongs survival in mice (Alexandrova et al., 2015). In another mouse study, the authors examined the GOF properties of two p53 mutants (R172H and R270H, corresponding to human R175H and R273H) expressed from endogenous p53 loci. The p53^−/−^ mice spontaneously developed more extensive tumors than the p53^−/−^ mice, including more cancers and more frequent endothelial tumors (Olive et al., 2004), which shows the biological effect of mutant p53 GOF. However, in a CRC mouse model carrying a p53 R270H mutation, p53^−/−^ mice showed tumor load, frequency of metastasis, and overall survival similar to that of p53^−/−^ mice that opposed p53 GOF (Tang et al., 2019).

### 5.2 Age-related secretory phenotype (SASP)

**FIGURE 2:**
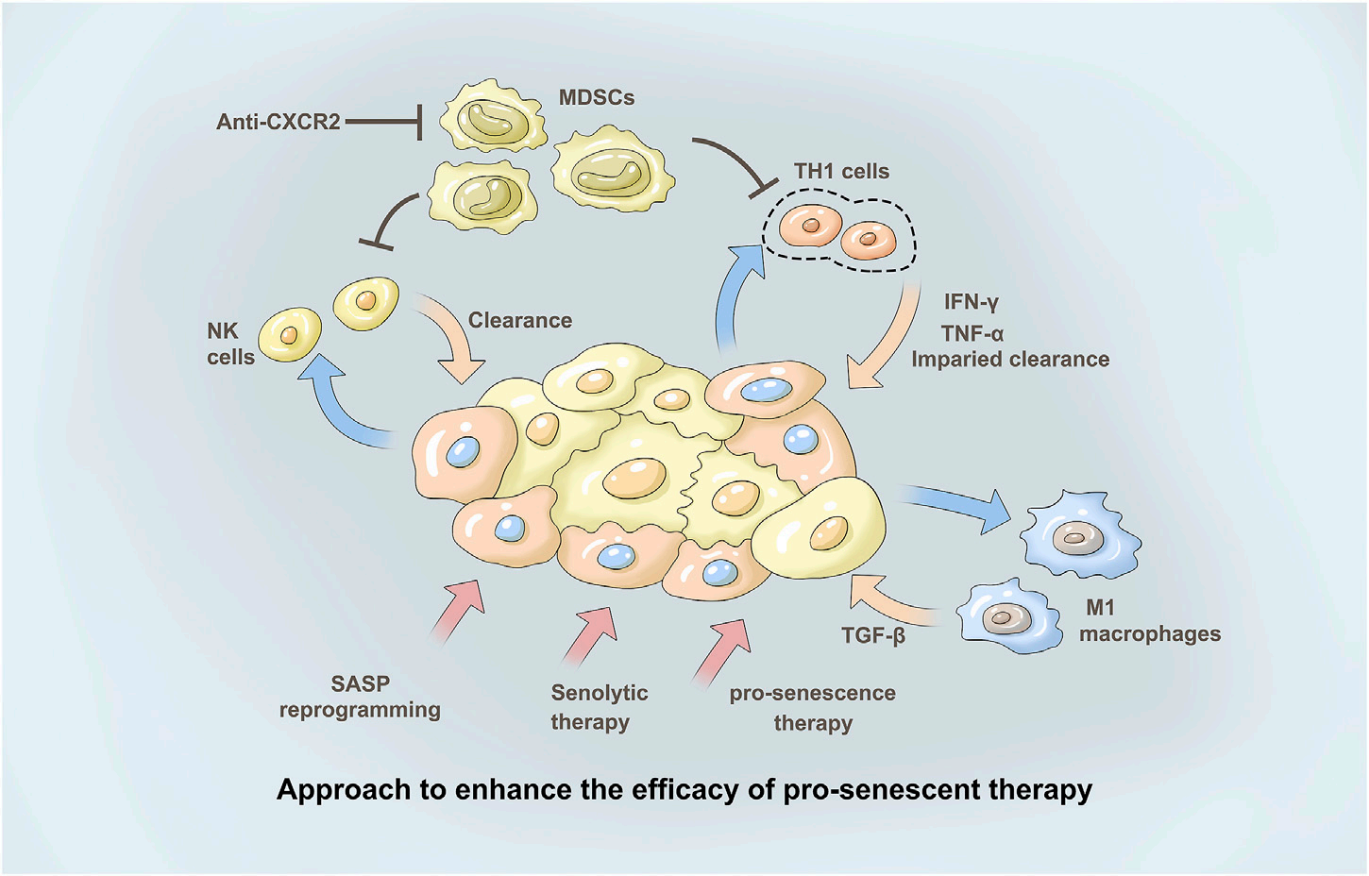
Approach to enhance the efficacy of pro-senescent the therapy. Immunotherapies may en-hance tumour clearance in tumours treated with pro-senescence therapies. Pharmacological reprogramming of the SASP may increase theanti-tumour immune response in tumours upon treatment with pro-senescence therapies. Senolytic therapies may remove senescence tumourcells in tumours where senescence surveillance is impaired, to avoid negative effects induced by the SASP. With immunosuppression of SASP secretion, the infiltration of NK cells decreased. At the same time, M1-type macrophages and their related secretory factors TGF-β as well as secretory factors IFN-γ and TNF-α of TH1 cells further play a role, thus promoting tumor treatment. The CXCR2 axis forms an immunosuppressive microenvironment involved in tumor immune regulation. Anti-CXCR2 treatment limits MD-SC recruitment in the tumour, favouring senescence induction and/or antitumour immunity.

Although senescent cells have many common characteristics, each senescent cell population shows different levels of cytokines, growth factors, and proteases, which is known as the age-related SASP (Cuollo et al., 2020). SASP secretion is essential for senescence function, and ILK or its downstream signaling partners, including PTEN, PI3K/Akt/mTOR, and NF-κB, may influence SASP regulation (Lopes-Paciencia et al., 2019; Almasan, 2021). This is accompanied by immunosuppressive SASP secretion, but upregulation of the immunostimulant SASP also occurs. This subsequently increases MDSC infiltration without CD4 T cells, CD8 T cells, and NK cells (Hinshaw and Shevde, 2019). This study also showed that SASP can be programmed by targeting STAT3 to suppress immunosuppressive secretions while maintaining immunostimulant secretions, resulting in a decrease in infiltrating MDSCs and an increase in CD4 T cells, CD8 T cells, B cells, and NK cells (Figure 2). Inhibition of ILK has been shown to block activation of NF-κB and inhibit production of TNF-α, IL-6, and IL-1β as well as infiltration of inflammatory cells in a mouse model of colitis (Figure 2). Therefore, ILK may influence secretion of SASP factors in the cancer background by regulating NF-κB (Almasabi et al., 2021).

### 5.3 Ki67

Ki67 is a cytonuclear protein that is also a proliferative marker. As senescent cells are marked by permanent exit from the cell cycle, they do not express Ki67, a nuclear antigen specifically related to cell proliferation (Yang et al., 2018). Many studies have confirmed that high expression of the Ki67 protein is closely related to the biological characteristics and prognosis of tumors (Menon et al., 2019). Therefore, Ki67 has become one of the most reliable indicators to detect the proliferation activity of tumor cells and has been used for routine detection in clinicopathological diagnosis to guide the selection of postoperative treatment and prognosis assessment. However, retrospective studies have also reported no direct correlation between the Ki67 expression level and the clinical outcome of CRC patients (Ma et al., 2020).

## 6 Mechanism of anti-senescence therapy in GI cancer

### 6.1 Clinical pro-senescence therapy

Cyclin D1 is the activator of CDK4 and CDK6. Cyclin D1-CDK4/6 directly phosphorylates, stabilizes, and activates the transcription factor FOXM1, which promotes cell cycle progression and protects cancer cells from senescence (Tchakarska and Sola, 2020). Cyclin D-CDK4/6 also phosphorylates and inactivates TSC2, a negative regulator of mTORC1, resulting in activation of mTORC1. Conversely, inhibition of CDK4/6 results in decreased mTORC1 activity and reduced protein synthesis in different human tumor cells. Cyclin D1-CDK4/6 also increases the catalytic activity of PRMT5/MEP50, reduces the level of MDM4 protein, and leads to activation of p53, ultimately blocking cell cycle progression (Montalto and De Amicis, 2020). CDK6 expression has been shown to be elevated and associated with poor prognosis in gastric cancer. The CDK4/6 inhibitor PD-0332991 promotes the apoptosis and senescence of gastric cancer cells and inhibits the migration and invasion of gastric cancer cells (Liu et al., 2022). In the NCT03446157 study, the CDK4/6 inhibitor palbociclib was used in combination with the CD73 inhibitor AB680, showing potential for antitumor efficacy in animal models of CRC (He et al., 2018; Noh et al., 2022).

p53 mutations associated with cancer are mainly divided into two categories: mutations involving DNA contact with amino acids, which have very little effect on structure; and conformational mutation, which can lead to large structural changes or even misfolding. Both mutant p53 and wild-type p53 can regulate the tumor microenvironment to inhibit or promote tumors (Wang et al., 2015). P53 inhibits tumor progression by controlling the composition of microRNAs carried by exosomes and the pattern of cytokine secretion, thereby maintaining the differentiated state of tumor-associated nerves, and inhibiting neutrophil infiltration, respectively (Zhou et al., 2021). Conversely, p53 mutants support tumor progression by regulating exosome content, causing macrophages to reprogram to the M2 state, resulting in a more favorable tumor microenvironment (Liebl and Hofmann, 2021) (Figure 2).

Cycloastragenol induces apoptosis and inhibits the proliferation of colon cancer cells by activating p53 (Park et al., 2022). Cancer cells also disrupt p53 signaling through deregulation of non-coding RNAs. Studies have shown that miR-1827 and miRNA-766, two microRNAs targeting MDM2 and MDM4, respectively, are often downregulated in CRC samples; hence, their reduced expression in CRC may lead to impaired p53 stability (Chen et al., 2019). Telomerase can be treated with vaccines that stimulate an immune response to surface hTERTs (Bernardes de Jesus and Blasco, 2013). Cancer cells process endogenous hTERT and present hTERT peptides on the cell surface *via* major histocompatibility complex (MHC) I and II molecules. hTERT vaccines usually contain enzymatic peptides that are injected into the dermis, where dendritic cells present antigens to CD4^+^ T-helper 1 (TH1) cells in the lymph nodes (Kailashiya et al., 2017). These hTERT-specific TH1 cells migrate to tumors, where they stimulate the activity of CD8^+^ T cells against hTERT-expressing cancer cells or directly kill cancer cells by releasing cytokines, FAS, or tumor necrosis factor-associated apoptosis-inducing ligand (TRAIL) apoptosis-inducing receptor interactions (Ahmed and Tollefsbol, 2003).

The CXCR2 axis forms an immunosuppressive microenvironment involved in tumor immune regulation (Korbecki et al., 2022). Studies to date on CXC-mediated formation of immunosuppressive cells that promote tumor growth include tumor-associated macrophages (TAMs), myeloid suppressor cells (MDSCs), regulatory T (Treg) cells, tumor-associated neutrophils (TANs), plasmacytoid dendritic cells (pDCs) and B cells (Figure 2). *In vitro* and *in vivo* experiments on a variety of tumors have proven that high expression of CXCR2/CXCL1-2-5-8 correlates with the invasion of TAMs, MSDCs, Tregs and TANs and plays a role in promoting tumors (Zhang et al., 2020).

### 6.2 Preclinical senescence promotion therapy

SIRT1 plays a complex role in the senescence process, and SIRT1 levels increase during senescence in response to increased oxidative stress and its deacetylation, thus reducing the regulatory effect of gene transcription. SIRT1 can regulate expression of ARHGAP5 and inhibit the migration and invasion of gastric cancer cells (Dong et al., 2018). SIRT1 expression is also significantly associated with shorter overall survival and relapse-free survival. It is an important prognostic index for patients with gastric cancer (Cha et al., 2009). Detection of SIRT1 gene expression in gastric epithelial cells can be used as a prognostic indicator for gastric cancer progression (Mohammadi Saravle et al., 2018). Catalpol mediated microRNA-34a can directly target and regulate SIRT1, and play a role in inhibiting the occurrence and development of CRC through the inhibition of SIRT1. PMID: 32323786. miR-373 can specifically target the 3′-UTR of SIRT1, reduce its expression in pancreatic cancer cells, and exert anti-proliferation and pro-apoptosis effects on pancreatic cancer cells. PMID: 34096221.

Attempts to directly target MYC have focused on the MYC\/MAX heterodimerization domain. A dominant-negative mutant MYC peptide (OmoMYC) that directly binds to MYC and eliminates MYC function has been developed. Several small-molecule inhibitors (e.g., 10075-G5 and 10058-F4) destroy MYC\/MAX dimerization and have been shown to reduce MYC activity. Other methods include disrupting MYC function with covalent inhibitors. Hypusinated EIF5A promotes the growth of colorectal cancer (CRC) cells by directly regulating MYC biosynthesis at specific paused motifs (Coni et al., 2020). lncRNA GLCC1 can be stabilized by direct interaction with the HSP90 partner, ubiquitination of c-Myc transcription factors, and reprogramming glycolytic metabolism to promote CRC occurrence. PMID: 31375671. Helichrysetin targets c-Myc, inhibits lactic acid production and efflux in gastric cancer MGC803 cells, and significantly inhibits the growth of MGC803 cells *in vitro* and *in vivo*. PMID: 34462561.

BET family proteins, especially BRD4, are important transcriptional and epigenetic regulatory factors that are closely associated with the progression of a variety of tumors, including colorectal cancer. Enhanced BRD4 protein stability weakens its binding ability to BET inhibitors, induces chromosome remodeling, promotes proto-oncogene enhancer activity, and ultimately leads to CRC resistance to BETis and increased tumor malignancy. To evaluate expression of pBRD4 in CRC, targeted activation of BRD4 provides a new direction for the treatment of CRC patients (Wang et al., 2021). Exosome circLPAR1 reduces BRD4 translation through METTL3-eIF3h interaction, thus inhibiting CRC occurrence. PMID: 35164758.

## 7 Conclusion

In conclusion, inducing senescence of tumor cells is of great significance for the treatment of GI cancer. Cellular senescence is a double-edged sword for cancer treatment. In future studies, we will further explore the molecular mechanism of tumor cell senescence, further enrich the therapeutic targets of GI cancer, find new treatment strategy to improve the therapeutic effect.
